# What is Behind Salmonella? Unusual Presentation in Two Pediatric Cases

**DOI:** 10.7759/cureus.8769

**Published:** 2020-06-22

**Authors:** Eman Nooreddeen, Aisha Mohammed Alemam, Atheer A Ghous, Abdulaziz A Abu Alnasr, Ghaya I Al-Qurainees

**Affiliations:** 1 Pediatric Nephrology, Prince Muhammed Bin Abdulaziz Hospital-National Guard Health Affairs-Almadinah, Madina, SAU; 2 Pediatrics, Prince Muhammed Bin Abdulaziz Hospital-National Guard Health Affairs-Almadinah, Madina, SAU; 3 College of Medicine, King Saud Bin Abdulaziz University for Health Sciences, Jeddah, SAU; 4 Pediatric Infectious Diseases, Prince Muhammed Bin Abdulaziz Hospital-National Guard Health Affairs-Almadinah, Madina, SAU

**Keywords:** pediatric, case, nephrology, infectious, pmbah, ngha, madina

## Abstract

Salmonella infection is an international public health concern. Salmonella organisms are Gram-negative bacilli that belong to the family Enterobacteriaceae, and more than 2500 Salmonella serovars have been described.

The most common clinical presentations of Salmonella infection are gastroenteritis, bacteremia, enteric fever, and chronic carrier state. Other rare extraintestinal infections include cellulitis, urinary tract infection, pneumonia, endocarditis, meningitis, brain abscess, and osteomyelitis.

Salmonella species resistant to first-line treatment such as ampicillin, chloramphenicol, and trimethoprim-sulfamethoxazole are referred to as multi-drug resistant. In recent years, extensively drug-resistant (XDR) Salmonella have appeared in Pakistan; XDR strains are resistant to multiple antibiotics, including first-line antibiotics, fluoroquinolones, and third-generation cephalosporins.

We report two interesting pediatric cases who presented with uncommon Salmonella infection. The first case is a child diagnosed with XDR typhoid fever in a country where the strain is not endemic. The second case is a child who presented with a Salmonella urinary tract infection who is otherwise immunocompetent and has no apparent underlying structural abnormalities of the urinary tract.

## Introduction

Salmonella infections are globally prevalent foodborne pathogens due to food and water contamination [1]. Salmonella organisms are Gram-negative bacilli that belong to the family Enterobacteriaceae. The genus consists of two main species: S. bongori and S. enterica. However, S. bongori does not infect humans. The S. enterica subspecies enterica, which is subdivided into more than 2500 serovars, is the causative pathogen in humans. Salmonella’s route of infection is ingestion of contaminated water and food [2].

The severity of Salmonella infections in humans varies depending on the serotype involved and the health of the human host. Children who are younger than five years, older adults, and immunocompromised patients (such as those with sickle cell anemia) are more susceptible to Salmonella infection than a healthy adult patient [3].

Clinical presentations of salmonella infections in humans vary between nontyphoidal salmonella (NTS) infections and enteric fever. NTS infections caused by S. enterica serotype Enteritidis and S. enterica serotype Typhimurium can range from asymptomatic carriage to gastroenteritis, urinary tract infection (UTI), bacteremia, and focal infections, including meningitis, brain abscess, and osteomyelitis. Enteric fever caused by Salmonella enterica serovars Typhi, Paratyphi (types A, B, and, rarely, C) can cause typhoid and paratyphoid fever, respectively. In these, the fever typically is gradual in onset, with headache, malaise, anorexia, and lethargy, abdominal pain, hepatomegaly, splenomegaly, dactylitis, and rose spots (present in approximately 30% of patients) [4].

In Saudi Arabia, Salmonella is one of the most important pathogens causing food poisoning, particularly in Umrah and Hajj seasons, and the prevalence of nontyphoidal Salmonella is more frequent than the typhoid type. S. Enteritidis was the most isolated serotype [1]. Antimicrobial resistance from antibiotic misuse in humans and animals could happen in Salmonella species S. enterica strains [5]. Strains that acquired a plasmid resistant to multiple antibiotics, including first-line antibiotics (i.e., chloramphenicol, ampicillin, and trimethoprim-sulfamethoxazole), fluoroquinolones and third-generation cephalosporins are referred to as extensively drug-resistant (XDR) enterica serovar Typhi (S. Typhi), which has emerged in Saudi Arabia [6].

The US Centers for Disease Control and Prevention ranks antibiotic-resistant S. Typhi as a serious threat that requires frequent monitoring and prevention to reduce the spread of the resistant strain [7]. In the past, the S. Typhi was treated routinely by chloramphenicol, ampicillin, and trimethoprim-sulfamethoxazole. However, a multi-drug-resistant (MDR) S. Typhi emerged in 1991 in India and nearby countries [8]. The MDR isolates are prevalent in parts of Asia and Africa. Reduced susceptibility to fluoroquinolones is also widespread, and sporadic cases of resistance to third-generation cephalosporins or azithromycin have also been reported [8].

The involvement of the genitourinary system by Salmonella infection is relatively rare, even in endemic areas [9,10]. Salmonella was first mentioned as a causative agent of UTI in 1946 by Seligman et al. [11]. There is a paucity of data in the pediatric literature concerning UTI and Salmonella [12-15]. UTI due to Salmonella could be related to hematogenous seeding from bacteremia or related to ascending infection through the urethra from contaminated stool [16]. Factors such as chronic illnesses, immunocompromised status, and underlying structural urinary tract abnormalities play an important role in the pathogenesis of UTI by Salmonella [13,17,18].

We report cases of two pediatric patients who developed uncommon Salmonella infection. The first patient presented with XDR typhoid fever after a visit to an area recognized as endemic to highly resistant Salmonella [6]. The second patient presented with Salmonella UTI but is otherwise immunocompetent and has no apparent underlying structural abnormalities of the urinary tract.

## Case presentation

Case 1

A healthy nine-year-old boy presented to the emergency department (ED) with abdominal pain. A few days prior to presentation, he visited the Shikarpur district, a province of Sindh in Pakistan. He had no associated fever, vomiting, diarrhea, or urinary tract symptoms. Surgical causes were excluded, and abdominal ultrasound was done, which showed right iliac fossa mesenteric lymph nodes. No treatment was administered, and the patient was discharged to home. Ten days later, he presented to the ED a second time with high-grade intermittent fever, fatigability, cough, and runny nose lasting two days. A few days prior to his second presentation to the ED, he had visited Mecca for Umrah accompanied by his family.

During his stay in Pakistan, he resided with local family members, and he had no contact with sick people. The source of food was from a local market, and the fruit and vegetables were not properly washed before consumption. Bottled water was used for drinking. His family did not seek medical advice before travel, and they did not receive typhoid vaccines nor malaria prophylaxis.

Physical examination upon admission revealed blood pressure at 117/70 mmHg, pulse rate of 149 beats/minute, respiratory rate of 30 breaths/minute, and temperature 39.3 °C. The patient was conscious, well-hydrated, and had good peripheral perfusion. His throat was congested with no follicles. He had no skin rash, meningeal irritation signs, or palpable lymph nodes. The abdomen exam showed hepatosplenomegaly; however, his abdomen was soft and non-tender with no guarding. The findings from the rest of the examination were unremarkable.

**Figure 1:**
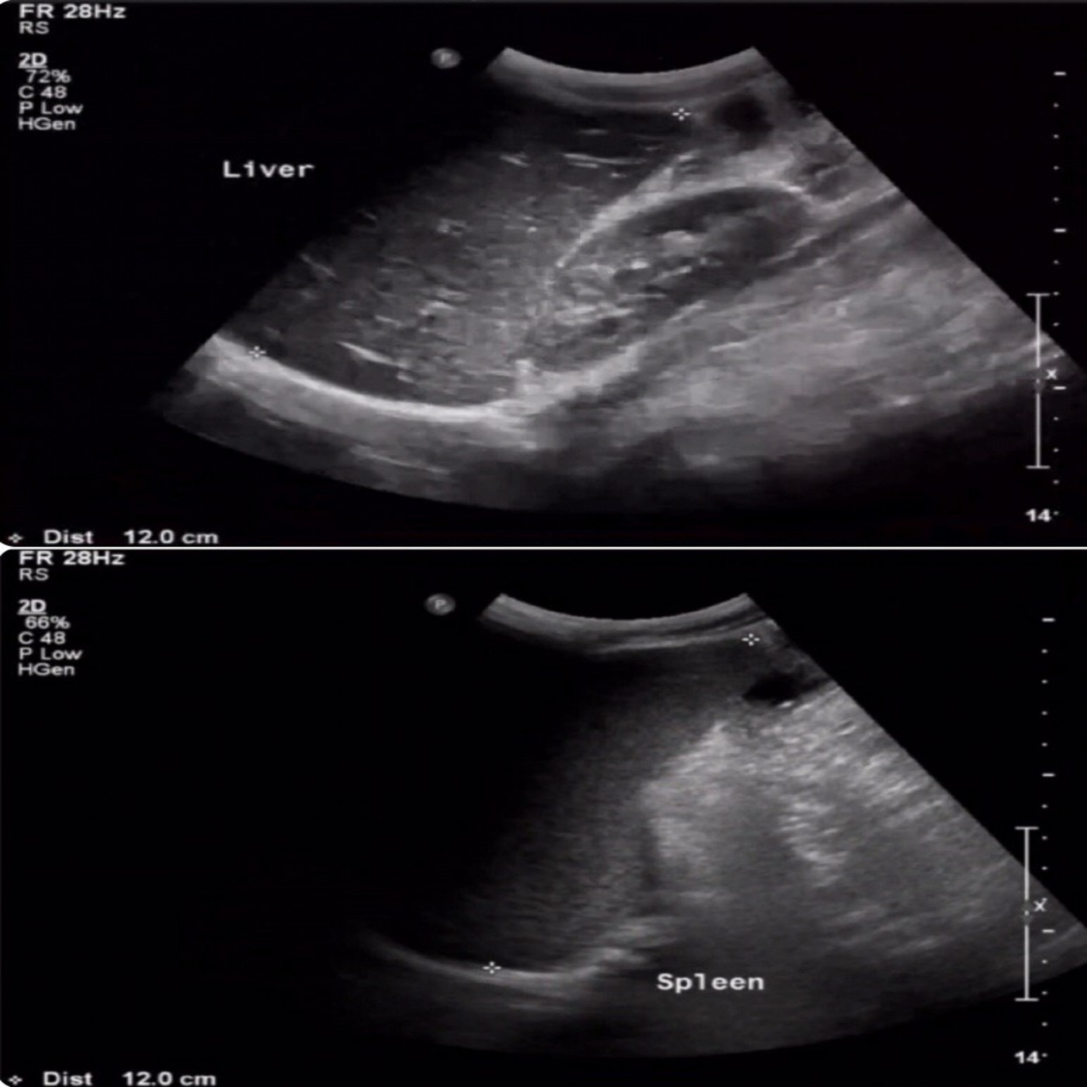
Abdominal ultrasound showing hepatosplenomegaly

The initial laboratory findings showed leukopenia, lymphopenia, and thrombocytopenia with a white blood cell count of 3.0 x 10^9^/L, lymphocyte count of 1.00 x 10^9^/L, and platelets count of 90 x 10^9^/L. His neutrophil count (2.72 x 10^9^/L) and hemoglobin level (13.4 gm/L) were within reference ranges. His C-reactive protein was elevated (92.1 mg/L). He had mildly elevated aspartate transaminase level (90 U/L), and his alanine aminotransferase level was within the reference range (33 U/L). His initial serum electrolyte levels were within reference range apart from mild hyponatremia (130 mmol/L). His peripheral blood smear was negative for blast cells, and it showed a low platelet count (with clumps). In the blood smear, we noted toxic granulation. He was negative for Malaria and Dengue. Stool culture showed no growth of Salmonella, shigella, or campylobacter, nor were ova or parasitic evidence present. Urine analysis findings were within reference ranges, and his urine culture was negative. An abdominal ultrasound showed that his liver was mildly enlarged (liver span: 12 cm) with no hepatic focal lesions or biliary dilatation. The spleen was mildly enlarged (size: 12 cm) with no focal lesions. We detected a mild amount of free fluid in the right iliac fossa. Blood culture was sent upon admission prior to starting antibiotics (Figure 1).

The patient had a throat swab taken for culture, and he was initially started on intravenous cefuroxime, treating a possible bacterial throat infection. However, the initial blood culture upon admission was positive for Gram-negative bacilli within the first 24 hours of incubation. Hence, cefuroxime was discontinued, a repeat blood culture was taken, and ceftriaxone was ordered. He received the first dose of ceftriaxone, and his care team consulted the infectious diseases team, who recommended changing to the use of meropenem empirically while waiting for the final blood culture results. The initial blood culture after three days of processing identified the isolate as Salmonella enterica serovar Typhi. Antimicrobial susceptibility test showed the isolate to be resistant to ampicillin, trimethoprim-sulfamethoxazole, ciprofloxacin, and ceftriaxone but susceptible to imipenem and meropenem.

He had two repeated blood cultures after the initial one that showed persistent bacteremia with the same isolate. On day 6 of therapy, a repeat blood culture was negative for bacterial growth. The patient received 14 days of meropenem therapy during which he showed progressive improvement of clinical and laboratory parameters. He was discharged for outpatient follow-up. Three consecutive stool cultures were taken 48 hours after discontinuation of antibiotic therapy to assess for Salmonella carrier state - all of which were negative.

Case 2

An 11-year-old boy brought to the ED with a history of sudden onset of bright red urine at the end of micturition (terminal hematuria) with small blood clots that started three hours prior to his presentation. He had associated painful micturition and urgency. There was no history of fever, flank pain, vomiting, trauma, body edema, or ingestion of medications or any type of red-colored food. Two weeks prior to his presentation, he had diarrheal illness without blood in his stool, which resolved spontaneously. He sought medical advice at that time, but no stool culture was taken. When he presented with blood in his urine, he had no current diarrhea. The child is an otherwise healthy boy with no history of recurrent urine or other body site infections in the past. He had no previous history of gross hematuria. No petechia, ecchymosis, or bleeding from other orifices. He underwent a routine circumcision as a neonate. His growth and development are appropriate for his age. There is no family history of hematuria, kidney or urological disorders, malignancy, bleeding, nor immunodeficiency disorders.

On examination, he looked well and hydrated. His body temperature was 36.8 °C, respiratory rate was 20 breaths/minute, heart rate was 76 beats/minute, and his blood pressure was 103/60 mmHg. He was not in pain. He had no edema or skin rash, and his abdomen was soft with no distension, tenderness, or organomegaly. Local examination revealed normal male genitalia with no local signs of infection or inflammation.

Urine analysis showed cloudy urine with a light orange color and urine pH of 8.5, specific gravity of 1.017, negative for glucose, negative for ketones, +3 blood, leukocyte esterase 500, negative nitrite, and +3 protein. Urine microscopy showed >30 red blood cells per high power field, five to 10 white blood cells per high power field.

His initial blood work showed prothrombin time was 12.3 seconds, partial thrombin time was 30.3 seconds, international normalized ratio of 1.03, white blood cell count of 9.8 x 10^9^/L with cell differential count of neutrophils 7.35 x 10^9^/L. His lymphocyte level was 1.60 x 10^9^/L, eosinophils were 0.04 x 10^9^/L, basophils were 0.03 x 10^9^/L, and monocytes were 0.70 x 10^9^/L. Blood chemistry showed serum sodium levels at 137 mmol/L, potassium at 4.1 mmol/L, chloride at 105 mmol/L, HCO3 at 23 mmol/L, blood urea nitrogen at 3.5 mmol/L, and creatinine at 52 μmol/L. The rest of the blood work showed a hemoglobin level of 148 g/L, platelet count of 356 x 10^9^/L, and blood glucose levels of 105 mmol/L.

A midstream urine sample was collected two different times, and both samples were sent for culture before starting antibiotics. Both cultures were incubated and grew more than 100,000 colony forming units per ml of Gram-negative bacteria. Afterward, the identification and susceptibility test showed Salmonella Nontyphi species. It was sensitive to ampicillin, ceftriaxone, ciprofloxacin, and trimethoprim-sulfamethoxazole. No further serotype identification was made due to a lack of test kit availability.

**Figure 2:**
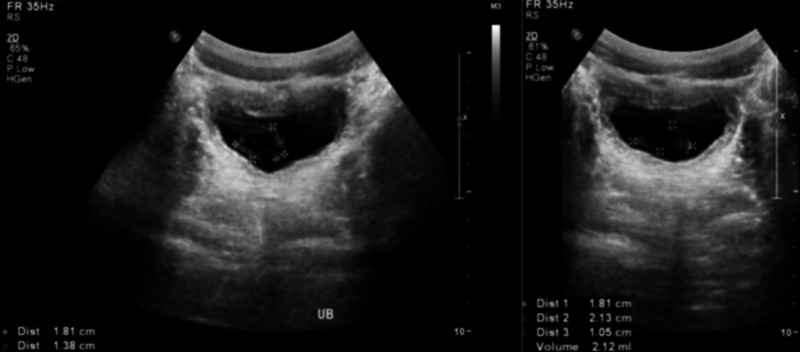
Renal ultrasound showing thickened urinary bladder wall

Renal ultrasound showed a urinary bladder wall thickness of 0.3 cm in a full bladder with luminal mobile echogenic foci at 2 cm x 1.3 cm. Otherwise, both kidneys were healthy in size, echogenicity, and corticomedullary differentiation with no hydronephrosis or ureteronephrosis (Figure 2).

The patient was admitted to the hospital and was started empirically on intravenous amoxicillin/clavulanic acid, which was continued for four days until the final urine culture result was assessed. Then, the patient was discharged home to complete three more days on the same antibiotic.

The patient was seen one month after discharge for follow-up; he was doing well with no gross hematuria episodes and no microscopic hematuria in urine analysis. The urology team saw him, and the plan was to consider cystoscopy if he became symptomatic again with another UTI. He was last seen seven months after the treatment of Salmonella UTI, and he was doing well with no recurrence of infection in the urinary tract.

## Discussion

Typhoid and paratyphoid fever occur globally, but primarily in South Asia, Southeast Asia, and sub-Saharan Africa, with both the most significant burden and incidence occurring in South Asia; 17 million cases estimated in 2015 [4]. If not treated, both typhoid and paratyphoid fever may be fatal; there were 178,000 typhoid and paratyphoid-related deaths estimated worldwide in 2015 [14].

The first-line medications for typhoid treatment are ampicillin, trimethoprim-sulfamethoxazole, and chloramphenicol. Strains of S. Typhi with resistance to these three antibiotics are considered MDR, and such isolates were first observed in the late 1970s and early 1980s [6]. The second-line for typhoid treatment is fluoroquinolones, ceftriaxone (a third-generation cephalosporin), and azithromycin (a macrolide). These second-line antibiotics became the preferred empirical treatment in regions with MDR infections [6].

Typhoid fever is endemic in Pakistan, and Salmonella resistance to first-line treatment as well as to quinolone represents a significant public health concern. Effective management of enteric fever should include control efforts directed towards water and food sanitation, hand hygiene as well as eliminating fecal carriers from the community. Since the emergence and spread of fluoroquinolone-resistant S. Typhi in Pakistan, the empirical treatment of choice for typhoid fever has been a third-generation cephalosporin given either via parenterally (for ceftriaxone/cefotaxime) or orally (for cefixime) [6].

In November 2016, the XDR strain of S. Typhi started to emerge from the Hyderabad district of the Sindh province in Pakistan. However, the pathogen remained sensitive to azithromycin and carbapenems [6].

To our knowledge, Case 1 represents the first pediatric case with XDR typhoid fever in Saudi Arabia, wherein a pediatric patient recently returned from Pakistan, where Salmonella is known to be endemic. The child did not receive a Salmonella vaccine. In this patient, the isolated strain of Salmonella was typhoid species Group C + D, which is similar to the most common strain reported in previous studies done in Saudi Arabia [1]. Moreover, the antibiotic susceptibility revealed this patient to have the XDR strain. This is contradictory to other studies that reported a high prevalence of resistance in nontyphoid Salmonella [1].

While the isolate was resistant to the first-line drugs, chloramphenicol and azithromycin susceptibility testing were not carried out because of internal microbiology laboratory policy in our center. Also, the strain was resistant to fluoroquinolones and third-generation cephalosporins, which classify it as XDR, sensitive only to meropenem and imipenem, which left limited treatment options. The case was effectively treated with meropenem [8].

Resistant enteric fever should be suspected in febrile patients from the Indian subcontinent and other Middle East countries [18]. Because of the emergence of ceftriaxone resistance in S. Typhi, culture- and sensitivity-guided treatment becomes important as empirical treatment with ceftriaxone is no longer reliable in these regions [18]. During the religious Umrah and Hajj seasons, large number of foreigners from all over the world visit the Kingdom of Saudi Arabia. Hence, it is challenging to ensure that visitors will follow the effective preventive measures. This will impose a possible introduction of MDR/XDR enteric fever from endemic countries.

Regarding the second patient who presented with UTI, Salmonella is a rare cause of UTI in children. It has been associated with a higher incidence in patients with chronic illnesses, structural abnormalities of the urological system, or immunosuppressive treatments [3]. Urologic abnormalities, including urolithiasis, chronic pyelonephritis, urethrorectal fistula, and retrovesicular fistula, can predispose a patient to nontyphoid Salmonella UTI [3]. Salmonella UTI was also reported in patients with immunocompromised states such as renal transplantation, human immune deficiency virus (HIV) infection with a CD4+ count of <100/mm^3^, chemotherapy, radiation therapy, and sickle cell disease [4]. Our case showed no evidence of immune deficiency by the lack of recurrent infections in the past nor structural abnormalities in the urological system by ultrasound screening.

According to our knowledge, only three cases are reported in the literature describing an immunocompetent child with a normally structured genitourinary system who developed UTI with Salmonella species.

Salmonella infects the urinary tract either by direct urethral invasion followed by ascending infection or by hematogenous spread [16]. The most common mechanism of infection has been documented as fecal contamination of the urethra, followed by retrograde urinary tract involvement [13].

## Conclusions

Although Salmonella UTI is a rare condition, it does occur in healthy children, especially in the presence of a history of gastroenteritis. The diagnosis of Salmonella UTI may signal the possibility of underlying structural abnormalities or immune deficiency disorders, especially if the patient has a relevant history or findings necessitating further workup.
